# Supplementary figures and images for: Interpreting T-cell search “strategies” in the light of evolution under constraints
Source: PLoS Comput Biol. 2023 Feb 27;19(2):e1010918. doi: 10.1371/journal.pcbi.1010918 (PMC9997883; doi:10.1371/journal.pcbi.1010918)

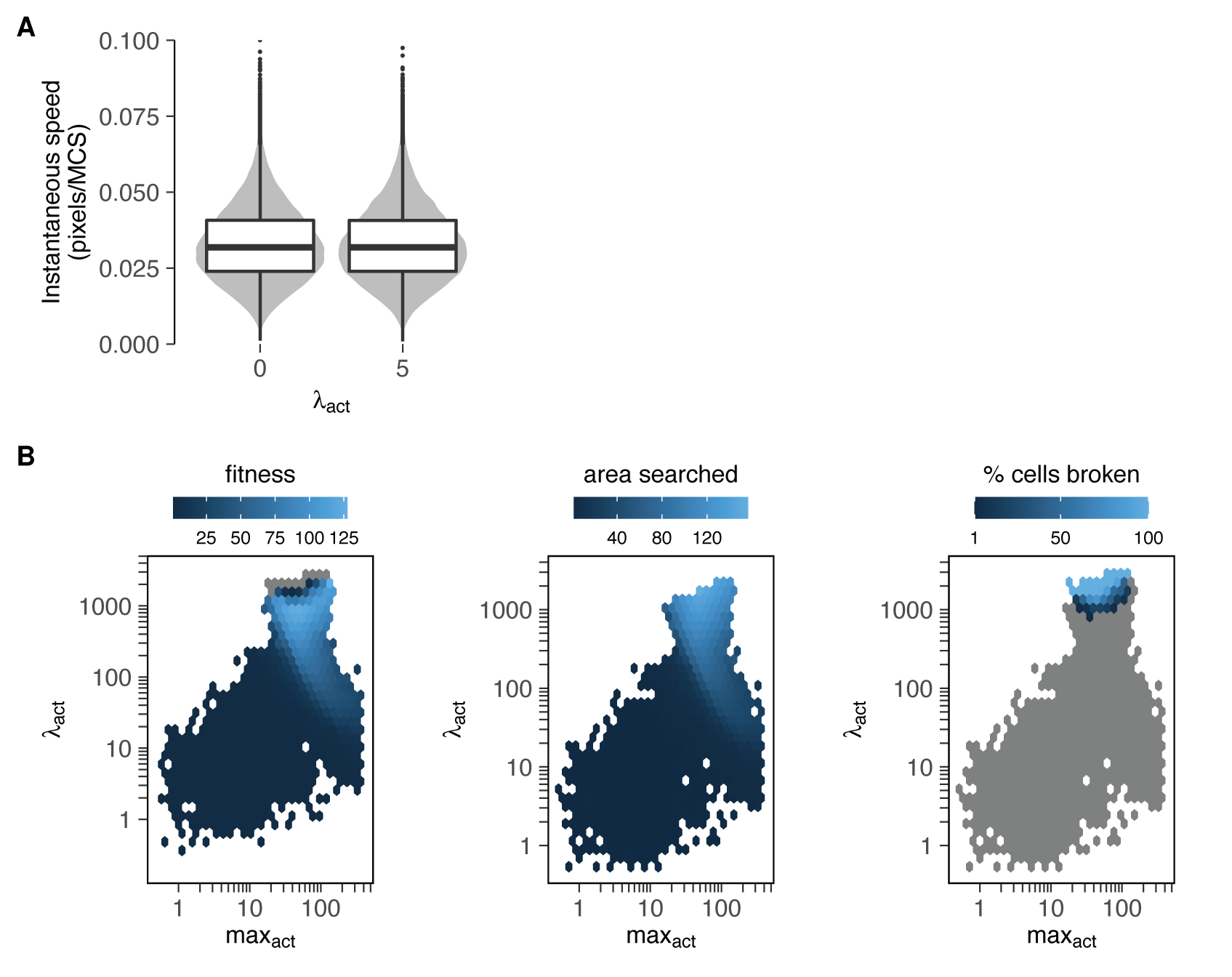

Supplement: S1 Fig — A: Act cells with maxact = 5 and λact = 5 cannot actively move. Distrubutions of instantaneous speeds equal those of control cells with λact = 0 (which cannot form protrusions by definition). B: Fitness landscape plots show mean fitness (area explored measured in the number of cell target areas of 500 pixels; broken cells have a fitness of zero), mean area searched by non-broken cells, and percentage of broken cells for different (maxact,λact) combinations. Gray fields represent a value of zero. (TIF) [file pcbi.1010918.s002.tif]

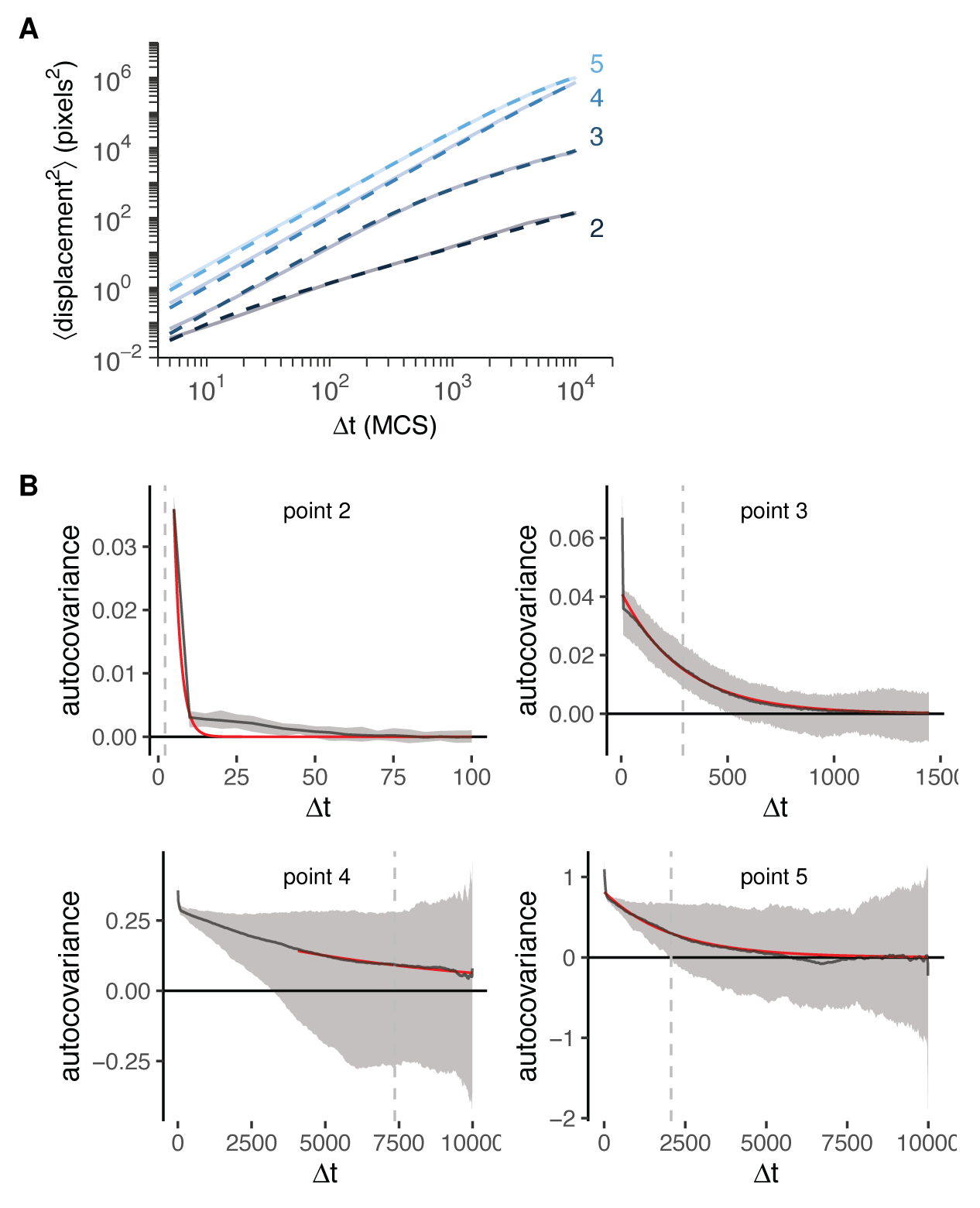

Supplement: S2 Fig — Motion was analysed for the points (2–5) along the evolutionary trajectory of Fig 3 (point 1 was skipped since at these parameters, cells do not yet move). A: Mean square displacement (MSD) curves of simulated tracks (solid) and the persistent random walk (P-RW) fit (dashed), for points 2–5 along the evolutionary trajectory. B: Autocovariance curves of the simulated tracks (mean ± interquartile range, gray) and an exponential decay fit (red, autocovariance ∼ exp -Δt/τ)). The dashed vertical lines represent the corresponding fitted value of τ, which is another measure of persistence time. (TIF) [file pcbi.1010918.s003.tif]

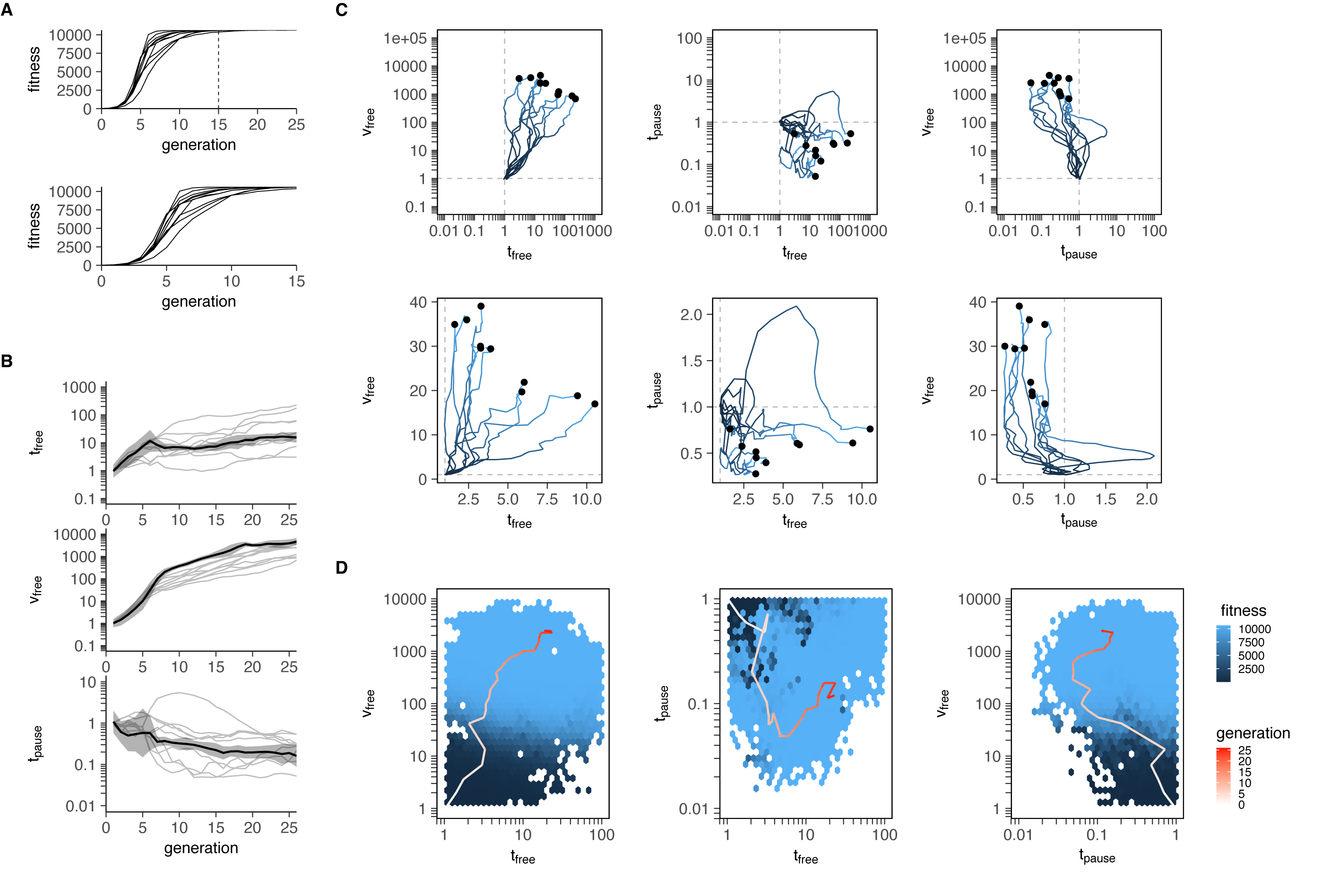

Supplement: S3 Fig — To illustrate the importance of a non-trivial mapping from model parameters to speed and persistence, we simulated evolution in the Beauchemin model of T-cell migration in the lymph node [4]. In this model, cells alternate between “runs” of duration tfree, during which cells move at speed vfree, and pauses of duration tpause, where cells do not move and can change direction. Thus, there is a more or less direct mapping from model parameters to migratory behavior, with vfree directly determining cell speed and tfree acting as a persistence time. At each step of fixed duration Δt, we check how much new explored area is within radius r = 13 of the cell’s current location. A: Fitness over generations (10 independent runs). After about 15 generations, the fitness stops increasing. This is because the fitness function measures area covered within radius r at every (discrete) time point where the cell is measured (determined by the time resolution Δt). Once the cell is fast and persistent enough that two subsequent circles do not overlap, and that it never returns to the same circle, it reaches a maximum fitness. This corresponds to a fitness plateau where cells can perform a random walk in parameter space. B, C: Evolution of the three independent model parameters over time. Independent runs follow the same trend (predictably increasing tfree and vfree, while decreasing tpause which does not help them explore area). But unlike with the Act-CPM, they do not converge to exactly the same end state (likely due to the fitness plateau observed in panel A). In panel C, trajectories are color-coded from dark (early generations) to light blue (later generations). D: Trajectory of one example run shown in context of the fitness landscapes for each possible pair of two parameters. This again shows that parameters first evolve towards fast, persistent motion. Once a good fitness is reached, they follow a random walk in the parameter space. Analogous to earlier figures, the “fitness” e [file pcbi.1010918.s004.tif]

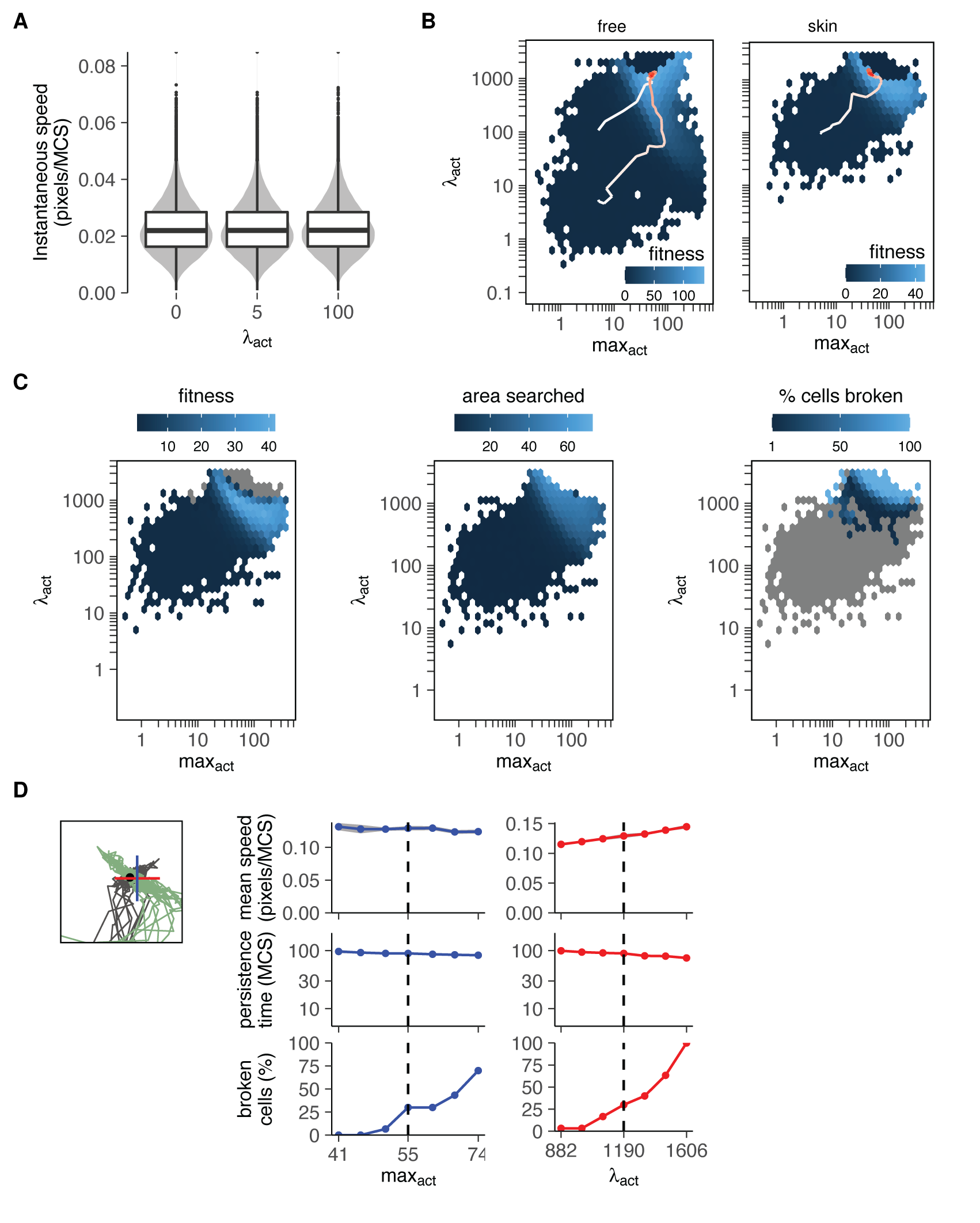

Supplement: S4 Fig — A: Cells with maxact = 5 and λact = 5 or λact = 100 cannot actively move in the rigid skin tissue. Distrubutions of instantaneous speeds equal those of control cells with λact = 0 (which cannot form protrusions by definition). B: Fitness landscape showing median fitness and example trajectories for cells evolved in an empty environment (“free”, two trajectories are shown with a different starting point) compared to cells evolved in stiff tissue (“skin”). C: Fitness landscape showing mean fitness, mean area searched, and percentage of broken cells (see also S1 Fig). D: Mean speed, persistence, and cell breaking of Act cells in simulated skin at parameters surrounding the evolved optimum (maxact = 55, λact = 1190). The square represents a zoomed version of Fig 4A showing this optimum. (TIF) [file pcbi.1010918.s005.tif]
