# Supplementary material for: Interpreting T-cell search “strategies” in the light of evolution under constraints
Source: PLoS Comput Biol. 2023 Feb 27;19(2):e1010918. doi: 10.1371/journal.pcbi.1010918 (PMC9997883; doi:10.1371/journal.pcbi.1010918)
Supplement: Attachment — Submitted filename: response-letter.pdf [file pcbi.1010918.s006.pdf]

Dear dr. Pitzer, dear reviewers,

We are grateful for the opportunity to submit a revised version of our attached manuscript for consideration at PLoS Computational Biology. We have carefully revised the manuscript to address the issues raised by the reviewers:

(I) An important criticism was that the claims of the paper were too strong. One simple model of evolution under constraints can of course not prove that evolution of search strategies in real T cells is impossible (points 2.2, 2.3, 2.4, 2.9), or works in the same way (1.2, 2.8, and 3.1). We completely agree and address this as follows:

- We never meant to claim that evolution of search strategies is impossible; indeed in our simple simulations we find that cells *can* evolve their migration patterns. Yet upon reading the manuscript again, we realized that some statements were worded too strongly and suggested that evolution is somehow impossible in the light of constraints. We apologize for this oversight and have rewritten the text (title, abstract, line 18-43,66,76-90,116-117,137,170-172,220-221,290-306[1]) to avoid this.
- It is well-known in the field of evolutionary biology that indirect genotype-phenotype mapping (and its effects on evolvability) can strongly affect *which* behaviors evolve. If this can reasonably be expected to hold for T-cell migration as well (which we show it can), this should be taken into account when interpreting migration patterns as search "strategies". However, we realized it was unclear in the original manuscript which concepts we introduce from the broader literature and which we directly showed ourselves. We have edited the text to clarify this (line 18-27,69-76).
- We added a discussion section (line 239-263) on model limitations and the generalisability of our results.

(II) Reviewer 2 agrees with us that "not all search strategies are evolvable", "an observed strategy should not be declared an evolutionary optimization without careful consideration of the context", and "most biological features reflect a compromise ... so that sufficing rather than optimizing should be the expectation". Although the reviewer calls this "not a surprise", we point out that these caveats are frequently ignored in the literature (see response to point 2.5 below).

(III) In response to point 2.7 below, we have edited the manuscript so as not to claim that tradeoff questions are impossible to answer in random walk models (see line 79-81,224). As the reviewer mentions, it is possible to impose constraints on random walk models as well. Yet we also note that this requires users to explicitly impose the relevant constraints on the model, and that often does not happen in practice.

To illustrate why this is important, we have added simulations with a model where cells can directly tune both speed and persistence (Figure S3, line 161-165,466-484). This shows that evolution can yield very different outcomes in a model that ignores constraints, highlighting once again that when it comes to interpreting search patterns, context is key.

(IV) The reviewers pointed out that the distinction between migration patterns evolved for search and those that arise through environmental constraints may not be so black and white. First, evolution takes place in an environmental context (point 2.6). Indeed, this is exactly why we feel this context should be taken into account when considering the evolutionary process, like we do here. Second, the environment may have co-evolved to facilitate motion (point 2.1), or cells may adapt to their environments without evolving their genotype (point 3.3). These are all very good points, and we have now added a discussion section on the possibility of adaptation and environmental co-evolution (line 265-289).

(V) We have edited the manuscript to provide clarifications as asked by the reviewers; specifically regarding the UCSP (point 1.1, line 35-36), the fitness (point 3.4, Figure 2A), the "peak" in the fitness landscape (point 3.2, line 147,157-160), and the comparison between evolved parameters (point 3.5, line 187-188,195-199).

We thank the reviewers for their input, which we feel has considerably improved the manuscript. We have considered and addressed every point (for details, see the point-by-point response below). We hope that with these revisions, the manuscript is now suitable for publication in PLoS Computational Biology.

Kind regards,

Johannes Textor and Inge Wortel

[1] line numbers refer to the pdf with changes tracked

Point-by-point response

1 Reviewer 1

This is an excellent paper that challenges the notion that T cell motility is evolutionarily optimized for particular goals relating to immune surveillance. Instead, the authors argue that constraints on cell movement imposed by the environment and the need to preserve membrane integrity result in patterns of movement that have previously been described as optional search strategies.

I believe this is a lovely example of how modeling can be used to simplify our thinking and pummel dogma. It emphasizes that optimality arguments are a logical and practical minefield, which – as the authors point out – has been a topic of debate in other fields, but so far not in this one. I think this paper will be an elegant contribution to Plos CB that is very much in the journal's scope.

I only have a couple of comments.

1.1.
(L26) I think it would be useful here to provide more detail regarding UCSP, rather than just citing ref. 13. it is an important concept and it would help to describe up-front how the basic result emerges.

Answer:
Thank you for pointing this out. We have added a sentence explaining the mechanistic basis of the UCSP in the introduction (line 35-36).

1.2.
It might be useful to highlight the potential limitations of the CP model. There is a tower of cards here – one can describe the genotype-phenotype map in terms of the CP model parameters, but this is a model itself – is it not possible that the true constraints may be entirely elsewhere? If this is the case, the utility of optimality arguments in complex biological system may truly be fruitless!

Answer:
This is a very good point. Our model illustrates how search strategies might evolve under realistic constraints, but is unlikely to precisely capture *all* constraints acting on cells in reality.

Interestingly, we do have reason to believe that some of the constraints captured in this particular model might apply to real cells as well, and might give rise to similar trade-offs as the ones we have described here. Of course, this is by no means a guarantee that our model is correct and that we can predict optimality and evolution correctly. But since our model captures the "local activation, global inhibition" principle that underlies more models of cell migration, it would be interesting to see if different encodings of this same principle would evolve in similar ways.

We have added a section to the discussion (line 239-263) on model limitations, where we also discuss to what extent the results in our model might apply to real cells.

2 Reviewer 2

This manuscript asks an important question: should we assume that movement patterns of T cells, often considered to reflect search strategies, indicate that those search strategies have evolved, or do they simply reflect movement constraints?

2.1.
The simulations show that a relationship between persistence and speed may arise due to movement constraints rather than an evolved optimization of persistence and speed to cover more area. This result is not surprising – the USCP can be observed without invoking evolution. It is also important to consider another possibility –the T cells and the structures that constrain them may have co-evolved so that T cells find their targets within the organism's structures efficiently. This is not necessarily the case, but the manuscript ignores that there may be co-evolution between T cells and the environments they navigate (both of course under evolutionary pressures from the host organism).

Answer:
Indeed, this is true: especially in the case of lymphoid organs, whose primary task is to facilitate immune cell interactions, there is a possibility that the tissue architecture and cell migration patterns may both have (co-)evolved to facilitate immune cell "traffic". For example, there have been many studies on the FRC network and its potential role in helping naïve T cells find their antigens inside the lymph node. However, some caveats apply:

1. The argument "the environment and cell motion patterns might have co-evolved for function X" is associated with the same logical issues as the argument "cell motion has evolved to optimize function X" – especially since co-evolution of cells and their environment is even more difficult than evolution of cells by themselves. This does not mean that such co-evolution is impossible, but as the reviewer also points out (point 2.5), context and alternative explanations should at least be considered. For example, it is always good to consider what kind of tissue architecture you might expect without invoking evolutionary optimisation, and to discuss to what extent it seems reasonable that the environmental structure would evolve in this way.
2. The latter is especially important in non-lymphoid organs. In organs such as the brain (considered by Harris et al, ref 24) or the skin (simulated here), it is harder to imagine that the environmental structure would evolve to optimize T-cell search: what is "optimal" for T-cell search might be undesirable for other reasons.

We agree with the reviewer that this discussion was missing from the manuscript, and have now added a discussion section on this topic (lines line 265-289).

2.2.
While it is logical that not all search strategies are evolvable through natural selection, it is unclear how that is relevant here. The possibility of environmental constraint determining movement patterns does not at all suggest that evolving search strategies is impossible.

Answer:
We thank the reviewer for pointing this out. We have carefully reviewed the manuscript and found that some of our statements were worded too strongly, suggesting that evolution of search strategies is impossible. This was not our intention and we apologize that this was unclear. Indeed, the presence of constraints and hurdles is no proof that evolution does not occur – and as the reviewer rightfully points out, this conclusion would be an overclaim.

In fact, even in our simulation we find that search strategies *do* evolve to a large extent, towards migration parameters that yield both high speed and persistence. But we also find that only parts of the observed migration pattern can be traced back to this optimization, with others instead arising from cell-intrinsic and environmental constraints. In our simulations, cells sometimes develop migratory patterns that are *not* beneficial for their searching task.

We have carefully revised the manuscript (title, abstract, line 18-43,66,76-90,116-117,137,170-172,220-221,290-306) to clarify that we are not claiming that evolution is impossible, but that caution is still required when interpreting observed patterns as "optimal".

2.3.
It is particularly misleading to suggest that the lack of speed or persistence genes hampers the ability of these traits to evolve. The genotype phenotype mapping does not require such a reductionist view. A wide range of search strategies have evolved in animal movement foraging and migration without a one-to-one mapping of genes coding for particular phenotypic traits.

Answer:
The reviewer is right that not having speed or persistence genes cannot “hamper” the ability of migratory traits to evolve – at least not in the sense of making this evolution impossible. However, the role of genotype-phenotype mapping has been extensively studied in evolutionary biology, where it is now well-known that indirect genotype-phenotype mapping can strongly affect *which* behaviors evolve. Thus, our argument is that it is important to learn from this and take these considerations into account when it comes to T-cell search strategies as well, because they may provide important context to the migration patterns that evolve.

See also the previous point; we have carefully revised the text so as to not suggest that genotype-phenotype mapping makes evolution impossible (see response to previous point and in particular line 25-32).

2.4.
The broad statements about the difficulty of evolving search strategies are highly speculative and not supported by the findings of the simulation which apply only to a very specific modeling context. In general, the paper seems to overreach and draw conclusions that are not warranted by the model results. However, the more modest claim that traits such as the USCP may be linked for genetic or physical reasons seems supported by the evidence cited.

Answer:
We completely agree and have taken care to address this; see the responses above.

2.5.
It is warranted to stated a caveat that an observed strategy should not be declared an evolutionary optimization without careful consideration of the context and other constraints that could have led to the observation.

Answer:
Yes, this is exactly the point that we tried to bring across. Unfortunately, this caveat is not always paid (sufficient) attention to in the literature, which is why we feel that this paper would be an important contribution to the T cell migration literature – alerting researchers to the crucial issue of evolution under constraints.

For example, Harris et al (ref 24) propose that a generalised Lévy walk in the brain “enables T cells to find rare targets with more than an order of magnitude more efficiency than Brownian random walkers” – but they determine search efficiency in a simulation without environmental structure, do not consider other mechanisms through which the observed migration statistics might arise, and do not ask which “baseline” migratory statistics should be expected within the tissue architecture of the brain. Fricke et al (ref 27) find that observed T-cell migration in the lymph is not the optimal strategy for searching new targets, but then conclude that the observed statistics “balance the extent and thoroughness of search”. But they do not consider the alternative explanation that T-cells in the lymph node may be moving this way due to mechanistic and/or environmental constraints. Shaebani et al (ref 28) show that speed-persistence coupling makes for more efficient searchers and suggest this “strategy” to be crucial for efficient immune responses – yet it is unclear what this means given that speed-persistence coupling is the baseline expectation when it comes to actomyosin-driven motion (refs 18-20, 13).

2.6.
It is not a surprise that most biological features reflect a compromise of multiple conflicting constraints and tradeoffs so that sufficing rather than optimizing should be the expectation. However, the question “Do we need an evolutionary explanation or is it a side effect?” is an oversimplification. The evolution of any trait is in an environmental context.

Answer:
Indeed, this should perhaps not be suprising – yet observed migration patterns are frequently discussed in the context of optimality (see response to previous point).

Note that if a migration pattern can be explained as a side effect of some mechanistic constraint, this is "absence of evidence" rather than "evidence of absence" (of evolution). Even if we don't "need" it, the evolutionary explanation might still hold. But if there is such an alternative explanation, we hope the reviewer will agree that more proof is needed before declaring a behavior the result of evolution; the alternative explanations should at least be properly discussed. This might involve a discussion on how the environment might have evolved to facilitate migration – but such a discussion should probably also critically reflect on the feasibility of such co-evolution (see also the response to point 2.1 above).

We addressed this in the revised manuscript, where we have taken care not to claim that evolution is impossible.

2.7.
The statement that it is impossible to answer tradeoff questions in random walk models is also an overclaim. Such models can take environmental features and constraints into consideration. Not necessary to negate the possibility in order to explain

Answer:
We agree and have revised the statements in line 79-81,224 to focus more on the features that a model should have: realistic constraints that emerge from the basic rules of the model. Indeed, random walk models that can realistically incorporate this characteristic would be just as suitable. However, we also point out that this has not often been done so far. We therefore now also added a simulation of evolution with a simpler model where cells can tune speed and persistence independently (Figure S3), showing that this affects the outcome of evolution.

2.8.
The choice of the Act-CPM model is a good one, but it is not the only possible model that can address the question of how the environment influences motion. CPM make a particular set of modeling choices to generate tradeoffs as cells move. The simulations nicely demonstrate how those tradeoffs lead to the USCP. However, the manuscript extrapolates broadly from this particular modeling choice, and that seems unwarranted.

Answer:
Again, we agree. Aside from carefully revising the text to prevent extrapolating too broadly (see earlier responses), we also added a discussion section to the manuscript that explicitly describes model limitations, and to what extent we might expect these conclusions to generalise (line 239-263).

2.9.
It is also not clear that the very small number of cells and replicated in the GA are sufficient to make general claims about what can evolve more generally.

Answer:
This is true, and it was not our intent to make explicit claims about what can or cannot evolve, which is indeed something we could never do in our simple simulation (or any simulation, for that matter).

Instead, our aim was to provide a baseline of what sort of behaviors *might* evolve under realistic constraints, and to illustrate that we should expect these behaviors to be affected by other factors than functional optimisation alone. The revised manuscript now hopefully makes this clearer; see also the response to the other points.

In summary, the model presents a valid criticism of prior work that tends to ignore the role of the environment in shaping T cell movement. The paper is well-written. The model is explained clearly, and the results show an existence proof that the USCP can emerge from constraints of the environment. However, this was already known in work cited in the manuscript. The manuscript should be more cautious in interpreting the results of these simulations to broader meaning without justification.

3 Reviewer 3

The manuscript entitled "Constraints and trade-offs shape the evolution of T cell search strategies" considers a computational model of cell crawling to study the relation between the evolution towards and optimal behavior based on a fitness depending on the maximal area covered for a single cell during a certain time. A genetic algorithm is included to modify parameter values to mimic evolution during 50 generations. Such mathematical exercise is done in order to check if evolution can trigger the different phenotypes. The methods of the study are correctly described in the manuscript and the results look interesting. However there are still open questions and comments which the authors have to address to consider the manuscript adequate for the journal.

3.1.
It is not clear for me if the research done is just an interesting mathematical exercise or if the conclusions can be extrapolated to the reality of living cells. I mean I understand that the authors perform simulations to show that it is possible and it implies that it may be also possible in living cells, however this is not a proof, and evolution may affect quite different in this case. The arguments in the manuscript are in my opinion still not clear.

Answer:
This is true, and it was not our intention to "prove" that cells cannot evolve their migration patterns at all, or that they behave in the exact same way as in our simulation. Instead, our point is that there are alternative explanations for some of the behaviors that have previously been interpreted as "optimal". More generally, we show that even if the "task" being optimized seems trivial, the evolved behavior can still be different than expected because of constraints acting on the cell.

If certain behaviors *could* arise through other mechanisms than evolutionary optimization as we show here (even if we are not yet sure whether this applies to real cells), we hope the reviewer agrees that this should at least be considered and discussed. Evolutionary optimisation may not be the only explanation for the behavior observed, and our main point is that this possibility should be considered.

However, we agree that this point was unclear and have revised the manuscript at several points to remedy this. See the revised title, abstract, introduction, and discussion (line 18-43,66,76-90,116-117,137,170-172,220-221,290-306), as well as the new discussion section on model limitations and generalisability of results (line 239-263).

3.2.
The virtual cells maximizes during the evolution process described in the manuscript the ares covered, however there is a lack of explanation of the particular situation where all the trajectories in the fitness space converge. I have understood there is a compromise between increase of the persistence of the motion and to reduce cell breakup.

Answer:
Indeed cells maximize the area covered, which they can do by maximizing their speed and persistence. However, while doing so, they must also ensure that they don't break (because that would give them a fitness of zero).

So essentially the cells are optimizing three things at once: going as fast as possible, going as straight as possible, and keeping the forces to a manageable level so as not to break. But the cells can only affect these things *indirectly* by tuning their λ_{act} and $\max_{\text{act}}$ values. Because of this, they cannot actually fulfil all these demands and have to compromise instead (for example because at some point they cannot further increase persistence without losing speed, or because they cannot go faster without breaking as we see in Figure 3). The point in the fitness landscape the cells converge to then reflects the best compromise. We have edited the text (line 147,157-160) to explain this better.

3.3.
On the other hand the motion is associated to a particular cell genotype, however a single cell can adopt different strategies depending on the conditions. For example, depending on the starving level, Dictyostelium discoideum

cells present different persistence and speed of the motion. Therefore it is not clear for me that the genetic information determines cell speed and persistence in T cells.

Answer:

In the case of T cells, it is indeed possible (and even likely) that cells adapt their migratory behavior depending on tissue (e.g. lymph node versus peripheral organs) and context (e.g. memory T cells versus naïve T cells). However, evolving to a state where the cell can adapt to context is even more difficult than the simple scenario simulated here, so it becomes even less likely that the cell is truly optimized for each context rather than finding a compromise. Even in our highly simplified evolutionary setup, non-trivial migration patterns already emerge without being optimal – and this is only more likely to happen when evolution becomes more difficult.

We do agree that this is important to consider and have added a discussion section on the role of adaptation in T-cell search (line 265-289).

3.4.
I wonder which is the area covered by a single cell for the different conditions. In figure 2 the scale of colors shows the fitness , however is it the area covered by the cell? It is proportional?

Answer:
Indeed the fitness is proportional to the area covered by the cell, but we normalized this by the (average) cell area to get a more general notion of “area explored” that is less dependent on the spatial resolution of our model. The only exception is that when cells break, we assign them a fitness of zero.

We apologize that this was unclear and have adapted Figure 2 to show our definition of “fitness”.

3.5.
Resulting optimized parameters depend on the original condition? I have summarized the results form the manuscript:

- *skin conditions (large lambda initial) max_act = 55, lambda_act = 1190*
- *free conditions (small lambda initial) max_act = 50, lambda_act = 1165*
- *skin conditions (small lambda initial) max_act = 50, lambda_act = 1190*

In the skin conditions changing the initial value of lambda gives rise to “very similar parameters” corresponding to the same lambda_act = 1190, and (slightly?) different value of max_act, however it is the same that in the case of free conditions. This is puzzling. Is the process optimizing the area covered or just increasing speed and persistence avoiding cell break up?

Answer:
Indeed, the process is optimizing area covered, which it does *by* increasing speed and persistence (since higher speeds increase the amount of space a cell can explore in a given time, and a higher persistence makes this exploration more efficient by limiting the amount of space that is “visited” more than once). However, cells optimize this area under the additional constraint that they must avoid breaking (if they do, their fitness is zero). So the answer is that the cells are doing all three: they are optimizing the area covered, which they can do *by* increasing speed and persistence. But they can only increase speed and persistence indirectly by adjusting the λ_{act} and max_{act} parameters, which at very high values can have the “side effect” of causing cell breaking. So the cells evolve to the point of the parameter landscape where they achieve the highest speed and persistence they can get without breaking. This is hopefully more clear in the revised manuscript (line 147,157-160).

With this in mind, we can interpret the results as follows. Cells evolve to the part of the parameter landscape with optimal “fitness”, which reflects a compromise between functional optimisation (area explored) and constraints on the cell (trade-offs between speed and persistence, trade-off with cell breaking). When cells evolve in the same environment, they experience the same fitness landscape, as is the case for the comparison between “free” cells

that evolve from either a low or high initial λ_{act} value. So if they still end up at slightly different parameters, this is because of the stochasticity of both the CPM itself and the evolutionary algorithm (provided that they have had enough evolutionary time to reach the optimum from their original starting value). Thus, the difference between these two runs is sort of a baseline for the variation in the evolved optimum as expected from the stochasticity of the simulation. The revised manuscript (line 187-188) now explains this better.

By contrast, in the comparison between free and skin cells, cells *do* experience a different fitness landscape (Figure S4) – although the fitness differences are relatively minor. Indeed, the evolved parameters were not much different, with a difference similar to that based on stochasticity alone (see above) – likely because the fitness landscape was not all that different.

To further make sure that these differences are indeed negligible, we added the panel in Figure 4C. This showed that these small parameters, by themselves, did not notably affect speed and persistence – which were affected much more by the environmental structure than by the parameters of the cell itself. So although the exact values differ between conditions, these seem to be stochastic variations in outcome arising from the stochasticity of the model and algorithm rather than a ”true” difference in optimized parameters. We have revised the manuscript (line 194-205) to make this clear.
